# Severe bleeding tendency caused by a rare complication of excessive fibrinolysis with disseminated intravascular coagulation in a 51-year-old Japanese man with prostate cancer: a case report

**DOI:** 10.1186/1752-1947-6-378

**Published:** 2012-11-06

**Authors:** Yoshihiro Wada, Mitsuhiro Uchiba, Yoshiaki Kawano, Nobuyuki Kai, Wataru Takahashi, Jiro Honda, Ken-ichiro Tanoue, Yoshihiro Maeda, Yoji Murakami, Masatoshi Eto, Takahisa Imamura

**Affiliations:** 1 Department of Urology, Faculty of Life Sciences, Kumamoto University, 1-1-1 Honjo, Kumamoto, 860-8556, Japan; 2 Department of Blood Transfusion and Cell Therapy, Kumamoto University Hospital, 1-1-1 Honjo, Kumamoto, 860-8556, Japan; 3 Department of Molecular Pathology, Faculty of Life Sciences, Kumamoto University, 1-1-1 Honjo, Kumamoto, 860-8556, Japan

**Keywords:** Castration-resistant prostate cancer, Disseminated intravascular coagulation, Excessive fibrinolysis, Low-molecular-weight heparin, Tranexamic acid

## Abstract

**Introduction:**

Disseminated intravascular coagulation causes thrombotic tendency leading to multiple organ failure and occurs in a wide variety of diseases including malignancy. Disseminated intravascular coagulation is a latent complication in people with prostate cancer.

**Case presentation:**

A 51-year-old Japanese man with advanced castration-resistant prostate cancer was admitted to our hospital because of extensive purpura and severe anemia. Prolonged plasma coagulation time, hypofibrinogenemia and normal platelet count suggested that a decrease in fibrinogen induced a bleeding tendency causing purpura. However, elevated plasma levels of thrombin-antithrombin complex, fibrin and/or fibrinogen degradation products and D-dimers, with positive fibrin monomer test, manifested disseminated intravascular coagulation and subsequent fibrinolysis. Plasma levels of thrombin-antithrombin complex, fibrin and/or fibrinogen degradation products and D-dimers decreased after administration of low-molecular-weight heparin. However, low fibrinogen and α_2_-antiplasmin levels were not improved and plasmin-antiplasmin complex did not decrease, which revealed excessive fibrinolysis complicated with disseminated intravascular coagulation. We suspected that prostate cancer cell-derived urokinase-type plasminogen activator caused excessive fibrinolysis. Administration of tranexamic acid for fibrinogenolysis was added together with high-dose anti-androgen therapy (fosfestrol) for prostate cancer. Thereafter, prostate-specific antigen and plasmin-antiplasmin complex decreased, followed by normalized fibrinogen and α_2_-antiplasmin levels, and the patient eventually recovered from the bleeding tendency. Immunohistochemical staining of the biopsied prostate tissue exhibited that the prostate cancer cells produced tissue factor, the coagulation initiator, and urokinase-type plasminogen activator.

**Conclusion:**

This patient with rare complications of disseminated intravascular coagulation and excessive fibrinolysis is a warning case of potential coagulation disorder onset in patients with prostate cancer. We propose that combined administration of tranexamic acid and low-molecular-weight heparin together with high-dose anti-androgen therapy is a useful therapeutic option for patients with this complicated coagulation disorder.

## Introduction

One of the lethal complications of patients with solid tumors is disseminated intravascular coagulation (DIC) in which microthrombi are formed by abnormal activation of the coagulation system. Although consumption of coagulation factors and activation of the fibrinolytic system are always observed, severe bleeding tendency and excessive fibrinolysis are rare in DIC. In prostate cancer (PC), the incidence of DIC complication is from 13% to 30% whereas that of bleeding symptoms is only 0.40% to 1.65% [1]. PC cells express tissue factor (TF) [2] that initiates the extrinsic coagulation pathway [3] and leads to DIC [4]. Moreover, PC cells produce urokinase-type plasminogen activator (uPA) [5] that converts plasminogen to plasmin: a fibrinolytic protease [6]. TF is the causative of DIC that is reported to be a sign of metastatic PC [7]. TF and uPA are associated with PC metastasis and angiogenesis as negative prognostic factors [2,8,9]. Thus, it is possible that PC cells induce simultaneously DIC by TF and fibrinolysis by uPA in the patient, especially at the advanced stage. Little attention has been paid to the potential pathogenicity of PC cells, particularly in fibrinolysis.

In this paper, we report a patient with metastatic PC who showed a severe bleeding tendency with hypofibrinogenemia by excessive fibrinolysis accompanied by DIC. To elucidate the pathophysiology of the coagulation disorder, we analyzed values of coagulation and fibrinolysis markers and examined the biopsied PC tissue for TF and uPA production. Furthermore, we propose an atypical (unusual) but appropriate treatment for such a patient.

## Case presentation

**Figure 1:**
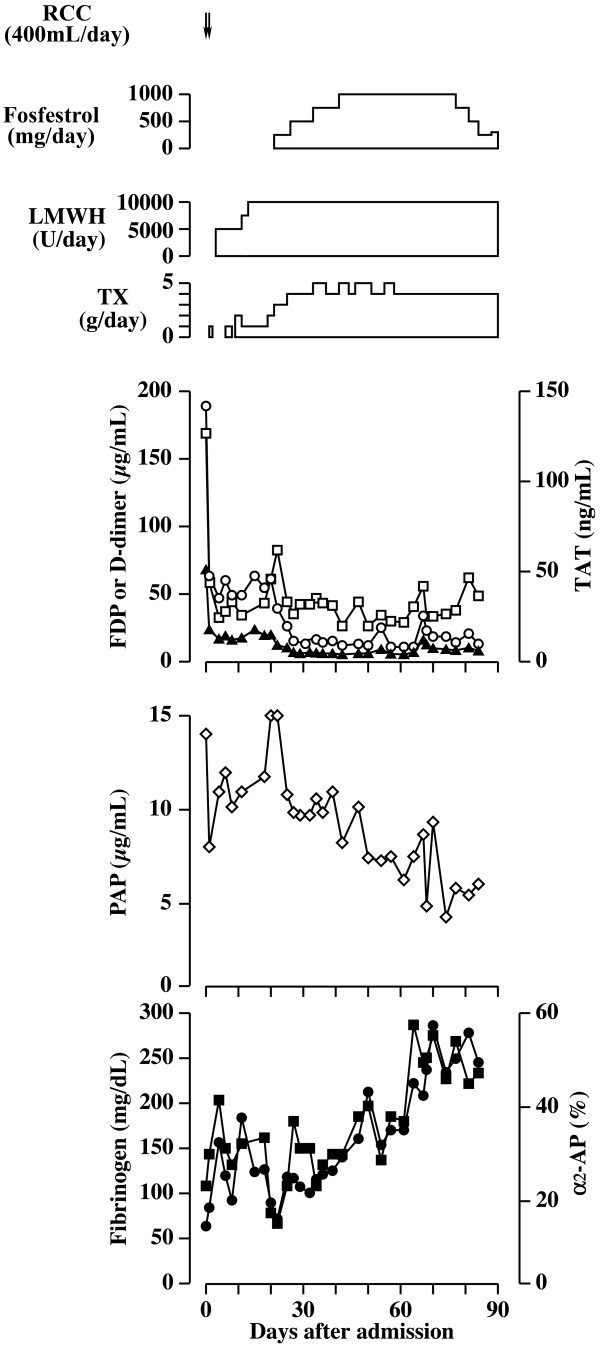
**Clinical course of the patient.** α_2_-AP: α_2_-antiplasmin; FDP: fibrin and/or fibrinogen degradation product; LMWH: low-molecular-weight heparin; PAP: plasmin-α_2_-antiplasmin complex; TAT: thrombin-antithrombin complex; TX: tranexamic acid. Open square: TAT; open circle: FDP; closed triangle: D-dimer; open diamonds: PAP; closed circle: fibrinogen; closed square: α_2_-AP.

A 51-year-old Japanese man who had advanced castration-resistant PC was admitted to our hospital because of extensive purpura and severe anemia. Although his platelet count was within the reference range and plasma clotting time was slightly prolonged, the fibrinogen level was low. Plasma levels of thrombin-antithrombin complex (TAT), fibrin and/or fibrinogen degradation products (FDPs), D-dimers and plasmin-antiplasmin complex (PAP) were high. Plasma α_2_-antiplasmin (α_2_-AP) level was low and a fibrin monomer test was positive (Table 1). These data indicated that he had DIC. Plasma TAT, FDPs, and D-dimers decreased (Figure 1) and platelet count increased after administration of low-molecular-weight heparin (LMWH). However, fibrinogen and α_2_-AP did not increase and PAP did not decrease (Figure 1). Bleeding diathesis continued whereas DIC parameters were improved by the treatment. To inhibit activation of fibrinolysis, administration of tranexamic acid was started together with LMWH. Then, PAP level decreased and levels of α_2_-AP and fibrinogen increased. Bleeding tendency disappeared. High-dose anti-androgen therapy (fosfestrol) for PC was also performed on day 20 for PC. Prostate-specific antigen level decreased to 482.4ng/mL.

**Table 1:** **Laboratory data on the first hospitalization day and relationship between fibrinogen, α**_**2**_**-antiplasmin, D-dimers/FDP ratio, thrombin-antithrombin complex, and plasmin-antiplasmin complex**

**Examination items**	**Value**	**Standard value**
Hemoglobin (g/dL)	5.6	14.0–17.7
White blood cells (/μL)	17,400	3500–8500
Platelet (/μL)	171,000	145,000–325,000
Prothrombin time (seconds)	17.6	11.0–15.2
APTT (seconds)	48.8	32.2–42.2
Fibrinogen (mg/dL)	62	150–450
Antithrombin (%)	65	81–121
Plasminogen (%)	76	70–111
α_2_-antiplasmin (%)	28	88–120
FDPs (μg/mL)	161.8	<5.0
D-dimers (μg/mL)	77.1	<1.0
Fibrin monomer test	Positive	Negative
TAT (ng/mL)	113.78	<3.0
PAP (μg/mL)	13.98	<0.8
PSA (ng/mL)	905	<4

*APTT* activated partial thromboplastin time, *FDPs* fibrin and/or fibrinogen degradation products *LMWH* low-molecular-weight heparin, *PAP* plasmin-antiplasmin complex, *PSA* prostate-specific antigen, *TAT* thrombin-antithrombin complex.

**Figure 2:**
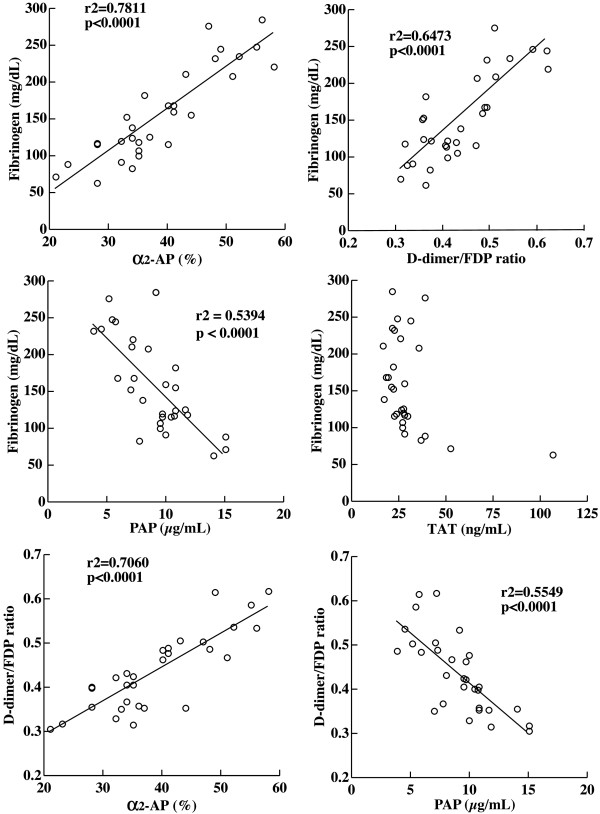
**Relationship between levels of fibrinogen, α**_**2**_**-antiplasmin, D-dimers/FDP ratio, thrombin-antithrombin complex, and plasmin-antiplasmin complex.** Pearson correlation coefficients (r) were used to determine the associations between the coagulation markers. α_2_-AP: α_2_-antiplasmin; FDP: fibrin and/or fibrinogen degradation product; PAP: plasmin-α_2_-antiplasmin complex; TAT: thrombin-antithrombin complex.

**Figure 3:**
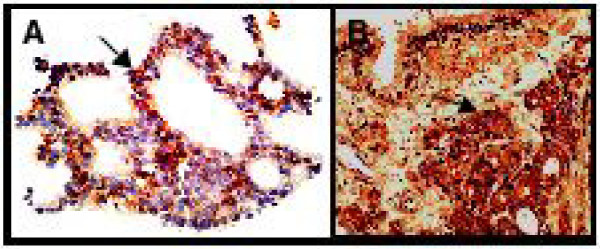
**Immunohistochemical staining for tissue factor (A, magnification ×200) or urokinase-type plasminogen activator (B, magnification ×100).** Arrows indicate positive cancer cells.

To investigate a correlation of coagulation and fibrinolysis markers, we analyzed the values of 31 plasma samples obtained during the 90-day hospitalization. As shown in Figure 2, the patient’s fibrinogen level correlated with levels of α_2_-AP (r^2^=0.7811, P<0.0001) and PAP (r^2^=0.5394, P<0.0001), and D-dimer/FDP ratio (r^2^=0.6473, P<0.0001). There was no correlation between levels of fibrinogen and TAT. The level of α_2_-AP (r^2^=0.7060, P<0.0001) correlated positively with D-dimer/FDP ratio and negatively with PAP level (r^2^=0.5549, P<0.0001). The prostate tissue biopsied for the initial diagnosis was immunostained using monoclonal antibodies specific for human TF [10] or uPA (Fuji chemical, Toyama, Japan). This analysis revealed that the cancer cells were positive for both TF and uPA (Figure 3).

## Discussion

We present a patient with PC who had a bleeding tendency of which the first sign was purpura. DIC is initiated by TF [4] with factor VIIa that activates the extrinsic coagulation pathway. TF is positive in more than 70% of human PCs and increase of TF in patients’ plasma is associated with patient poor prognosis [8]. The TF expression in the PC cells of this patient (Figure 3) suggests DIC induction by the cancer-derived TF. Coagulation activation results in fibrinogen consumption to some extent but the severe hypofibrinogenemia seen in the present case is unusual in DIC [11]. This was supported by no correlation between fibrinogen and TAT levels (Figure 2). By LMWH administration, levels of FDP, D-dimer and TAT decreased but the fibrinogen level was not improved (Figure 1). These results indicated that hypofibrinogenemia was not induced by DIC alone. Under normal conditions, plasmin degrades only stable fibrin (cross-linked fibrin) and is immediately inhibited by α_2_-AP in circulation. If the plasma α_2_-AP level is below 60% of the normal level, α_2_-AP is unable to control plasmin sufficiently, thus plasmin also degrades fibrinogen, fibrin monomers and unstable fibrin in addition to stable fibrin [12]. The high PAP and low α_2_-AP levels at the hospitalization day 1 (Figure 1) indicated consumption of α_2_-AP by forming complexes with increased plasmin. Plasmin generates D-dimers only from stable fibrin, whereas FDP derives from either fibrinogen or any forms of fibrin. Accordingly, the decrease in D-dimers/FDP ratio indicated a relative increase in fibrinogenolysis that could lead to hypofibrinogenemia. By the treatment with tranexamic acid, the low fibrinogen level elevated in correlation to the increase in D-dimers/FDP ratio and α_2_-AP level and to the decrease in PAP level. It is probable that the severe hypofibrinogenemia in this patient was caused by fibrinogenolysis due to excess plasmin production. The low plasma α_2_-AP level and high plasma PAP level agreed with the fibrinogenolysis observed in a patient with metastatic PC [13]. Bleeding diathesis of this patient was improved by administration of tranexamic acid in relation to elevation of α_2_-AP level, suggesting that excessive fibrinolysis induced bleeding diathesis. Platelet and coagulation factors, such as factor VIII and IX, are important in normal hemostasis. From data that the platelet count was within the normal range and clotting time was slightly prolonged, it is unlikely that bleeding diathesis was caused by thrombocytopenia or consumption of coagulation factors. The result that fibrinolysis activation was not improved by anticoagulation therapy alone, suggests an involvement of the primary fibrinolysis, at least partially, in the bleeding diathesis. The plasma uPA level was not measured, but uPA expressed in PC cells probably contributed to excessive fibrinolysis.

Unfractioned heparin has been used for treating patients with DIC complicated with cancer and shown to prolong their survival. Tranexamic acid inhibits plasminogen activation, thereby causing thrombotic tendency in typical DIC cases [14], therefore, this agent is usually contraindicative for DIC patients. However, in this case, together with LMWH for DIC, we administered tranexamic acid to the patient who manifested excessive fibrinolysis, and successfully controlled the complicated coagulation disorder. In such a case, tranexamic acid is potentially available for treatment of excessive fibrinolysis.

## Conclusion

A PC cell itself may often be overlooked as a causative of coagulation disorder because of the very low incidence of overt DIC in PC [1]. Even if a clinician notices DIC in such a patient, he or she may erroneously consider that any changes of coagulation markers are caused solely by DIC. Accordingly, such a patient may receive only heparin infusion, which would not adequately meet the patient’s bleeding tendency accompanied by excessive fibrinolysis induced by uPA secreted from PC cells. Although it is widely known that uPA plays important roles in PC cell invasion and proliferation [9,15], the classical aspect of uPA as an activator of the fibrinolytic system may paradoxically not be easily conceivable in patients with PC with bleeding disorder. Therefore, it is important to bear in mind that PC cell uPA can generate plasmin that degrades fibrinogen, leading to hypofibrinogenemia and resultant bleeding tendency. The present case of a rare complication of excessive fibrinolysis raises awareness of a potential fibrinolytic system being activated in patients with PC. Hence, we hereby propose the treatment of combined administration of tranexamic acid and LMWH together with high-dose anti-androgen therapy for a patient with PC with fibrinolysis coincident with DIC.

## Consent

Written informed consent was obtained from the patient for publication of this case report and accompanying images. A copy of the written consent is available for review by the Editor-in-Chief of this journal.

## Competing interests

The authors declare that they have no competing interests.

## Authors’ contributions

YW, NK, WT, KT, JH, YM and YM examined and treated the patient with PC. MU, YK and ME analyzed and interpreted the patient’s data. TI performed immunohistochemistry and supervised this report including manuscript revision. All authors read and approved the manuscript.

## References

[B1] Smith JA Jr Soloway MS Young MJ Complications of advanced prostate cancer Urology 1999 54 8 14 10.1016/S0090-4295(99)00448-3 10606278

[B2] Abdulkadir SA Carvalhal GF Kaleen Z Kisiel W Humphrey PA Catalona WJ Milbrandt J Tissue factor expression and angiogenesis in human prostate carcinoma Hum Pathol 2000 31 443 447 10.1053/hp.2000.6547 10821491

[B3] Nemerson Y Tissue factor and hemostasis Blood 1998 71 1 8 3275472

[B4] Levi M Disseminated intravascular coagulation Crit Care Med 2007 35 2191 2195 10.1097/01.CCM.0000281468.94108.4B 17855836

[B5] Cozzi PJ Wang J Delprado W Madigan MC Fairy S Russell PJ Li Y Evaluation of urokinase plasminogen activator and its receptor in different grades of human prostate cancer Hum Pathol 2006 37 1442 1451 10.1016/j.humpath.2006.05.002 16949925

[B6] Castelline JC Violand BN The mechanism of activation of human plasminogen by urokinase J Biol Chem 1976 251 3906 3912 132442

[B7] Duran I Tannock IF Disseminated intravascular coagulation as the presenting sign of metastatic prostate cancer J Gen Intern Med 2006 21 C6 C8 10.1111/j.1525-1497.2006.00506.x 17026724 PMC1831660

[B8] Akashi T Furuya Y Ohta S Fuse H Tissue factor expression and prognosis in patients with metastatic prostate cancer Urology 2003 62 1078 1082 10.1016/S0090-4295(03)00768-4 14665359

[B9] Pulukuri SM Gondi CS Lakka SS Jutla A Estes N Gujrati M Rao JS RNA interference-directed knockdown of urokinase plasminogen activator and urokinase plasminogen activator receptor inhibits prostate cancer cell invasion, survival, and tumorigenicity in vivo J Biol Chem 2005 280 36529 36540 10.1074/jbc.M503111200 16127174 PMC1351057

[B10] Imamura T Kaneda H Nakamura S New functions of neutrophils in the Arthus reaction: expression of tissue factor, the clotting initiator, and fibrinolysis by elastase Lab Invest 2002 282 1287 1295 10.1097/01.lab.0000032374.21141.15 12379763

[B11] Levi M de Jonge E van der Poll T ten Cate H Disseminated intravascular coagulation Thromb Haemost 1999 82 695 705 10605770

[B12] Okajima K Kohno I Soe G Okabe H Takatsuki K Binder BR Direct evidence for systemic fibrinogenolysis in patients with acquired alpha 2-plasmin inhibitor deficiency Am J Hematol 1994 45 16 24 10.1002/ajh.2830450104 8250008

[B13] Okajima K Kohno I Tsuruta J Okabe H Takatsuki K Binder BR Direct evidence for systemic fibrinogenolysis in a patient with metastatic prostatic cancer Thromb Res 1992 66 717 727 10.1016/0049-3848(92)90047-E 1519230

[B14] Koo JR Lee YK Kim YS Cho WY Kim HK Won NH Acute renal cortical necrosis caused by an antifibrinolytic drug (tranexamic acid) Nephrol Dial Transplant 1999 14 750 752 10.1093/ndt/14.3.750 10193833

[B15] Festuccia C Dolo V Guerra F Violini S Muzi P Pavan A Bologna M Plasminogen activator system modulates invasive capacity and proliferation in prostatic tumor cells Clin Exp Metastasis 1998 16 513 528 10.1023/A:1006590217724 9872599

